# FixItFelix: improving genomic analysis by fixing reference errors

**DOI:** 10.1186/s13059-023-02863-7

**Published:** 2023-02-21

**Authors:** Sairam Behera, Jonathon LeFaive, Peter Orchard, Medhat Mahmoud, Luis F. Paulin, Jesse Farek, Daniela C. Soto, Stephen C. J. Parker, Albert V. Smith, Megan Y. Dennis, Justin M. Zook, Fritz J. Sedlazeck

**Affiliations:** 1 Human Genome Sequencing Center, Baylor College of Medicine, Houston, TX USA; 2 Department of Biostatistics, University of Michigan School of Public Health, Ann Arbor, MI USA; 3 Department of Computational Medicine and Bioinformatics, University of Michigan, Ann Arbor, MI USA; 4 Genome Center, MIND Institute, Department of Biochemistry and Molecular Medicine, University of California, Davis, Davis, CA USA; 5 Material Measurement Laboratory, National Institute of Standards and Technology, Gaithersburg, MD USA; 6 Department of Computer Science, Rice University, Houston, TX USA

**Keywords:** Reference, GRCh38, T2T-CHM13, Variant, SNV, INDEL, Medically relevant genes, Remapping, GIAB, eQTL

## Abstract

The current version of the human reference genome, GRCh38, contains a number of errors including 1.2 Mbp of falsely duplicated and 8.04 Mbp of collapsed regions. These errors impact the variant calling of 33 protein-coding genes, including 12 with medical relevance. Here, we present FixItFelix, an efficient remapping approach, together with a modified version of the GRCh38 reference genome that improves the subsequent analysis across these genes within minutes for an existing alignment file while maintaining the same coordinates. We showcase these improvements over multi-ethnic control samples, demonstrating improvements for population variant calling as well as eQTL studies.

**Supplementary Information:**

The online version contains supplementary material available at 10.1186/s13059-023-02863-7.

## Background

The identification of genetic variation in individuals and populations is essential for all genomic analyses to answer questions related to evolution, diversity, diseases, and biological processes in general [1–3]. To identify variation, sequences are typically mapped to a reference genome [4, 5], though de novo genome assembly approaches using long reads are advancing rapidly [6, 7]. Typically, in both cases, methods compare to a single reference genome to form a unified coordinate system that enables the comparison of differences across multiple projects and thus enables novel insights. For humans, we have had a reference genome since the Human Genome Project released its first version in 2001 [8]. Since then, multiple updates have been made with the current version (GRCh38) slowly being adopted over the last decade [9]. Most recently, the Telomere-to-Telomere (T2T) Consortium released a new complete human genome reference (T2T-CHM13) [10]. Though initial studies show promising results [11], T2T-CHM13 currently lacks many of the resources and annotations that exist for GRCh38, likely hindering the community’s transition to this new reference.

Over the past years, it has become clear that a reference genome cannot be a single best representation of the human population or any given species, but it eases the comparison across studies and holds the promise of curated resources [12]. As such, the human reference genome never included all major alleles or all minor alleles from a population, but rather a mix of haplotypes from multiple individuals [9]. Nevertheless, multiple updates have corrected errors, added alternate loci, and improved certain regions of the genome to make the representation more complete and, thus, improve variant calling and comparison. Most recently, our work identified remaining issues with the most commonly used reference genomes (GRCh37+38), where certain regions of the genome have been duplicated along chromosome 22 [13]. These include at least three medically relevant genes for inherited diseases, as well as one relevant for somatic variant calling [14]. Continuing this work together with the T2T group revealed other artifacts along GRCh38, including additional false duplications and missing copies (or collapses) of segmental duplications [11]. As these regions also impact medically relevant genes, we are eager to correct them and improve mapping and variant calling.

In this work, we focus on providing a solution for these reference issues to improve variant calling across 9.24 Mbp of the genome including 33 protein-coding genes including 12 highly relevant medical genes. To achieve this, we propose a modified GRCh38 reference that includes several masked regions as well as newly introduced decoy contigs. Using this reference, we demonstrate improvements in mapping and single-nucleotide variant (SNV) calling across different ancestries. In addition, we propose a rapid, localized remapping framework that improves the alignment of short or long reads across targeted regions and provides a modified alignment (BAM/CRAM) file that can be used for subsequent variant identification. This approach improves accuracy not only for human individuals of European ancestry, HG002 (Genome in a Bottle [GIAB] NIST Reference Material) [15], but also for more ancestrally diverse groups. Furthermore, we assess the benefits not only for whole genome and exome sequencing of short and long reads but also for RNA sequencing analysis, making this an important analytical change for many studies to come. Maybe even more importantly, we highlight its improvements across different human ancestries such as African, European, and Asian populations. Thus, we show clear improvements for these genes across the 3202 samples from the 1000 Genomes Project (1KGP) [16] discovering novel alleles across these important genes and regions. We further investigate if these SNV improvements have a significant impact on phenotypic traits. Lastly, we highlight the importance of these introduced changes by showing improvements for eQTL studies. Altogether, we demonstrate the importance of the newly introduced changes to GRCh38 itself and a computationally efficient solution to improve existing mapped genomic (BAM/CRAM) data to identify genomic variations at accuracy and scale across these important genes.

## Results

### Identification of GRCh38 errors

**Fig. 1:**
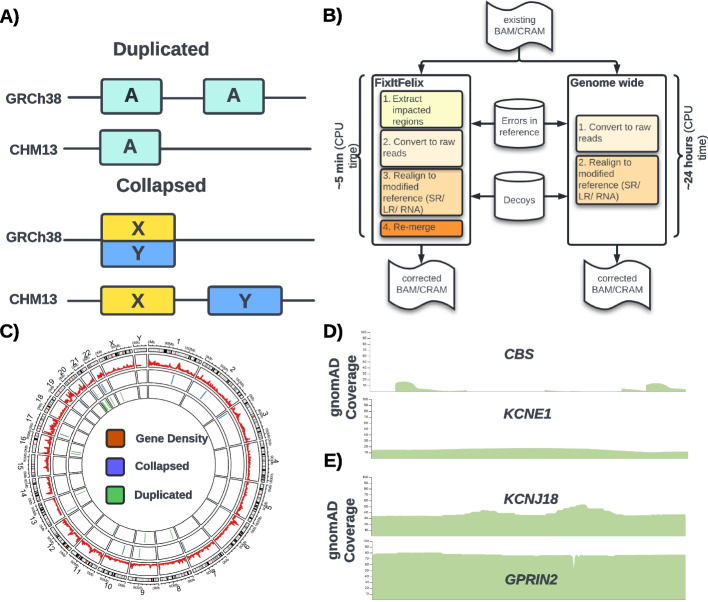
Falsely duplicated and collapsed regions of GRCh38 reference. **A** Cartoon showing the duplicated and collapsed errors. For duplicated errors, one extra copy that is absent in T2T-CHM13 reference is present in GRCh38. For collapsed regions, the two separate copies are merged into one region. **B** Our pipeline (FixItFelix), shown on the left side of the figure, extracts the sequences from impacted regions and remaps them to the modified reference for subsequent analysis. This takes only 4~5 min as compared to >24 h when remapping all sequences to the modified reference genome (i.e., global realignment, shown on the right side). **C** Twelve wrongly duplicated regions that include 22 protein-coding genes, 18 pseudogenes and a total 2,032,012 bp (1,021,203 bp of correct regions, and 1,010,809 bp of false duplication); 9 collapsed regions that include 9 genes and total 843,139 bp. **D** gnomAD track showing lower than normal whole genome sequencing (WGS) coverage of falsely duplicated genes (*CBS* & *KCNE1*). **E** gnomAD track showing higher than normal WGS coverage of collapsed genes *KCNJ18* and *GPRIN2* where one or more paralogs are missing

From our previous work [11], we identified errors in the GRCh38 reference genome, including (i) 1.2 Mbp of falsely duplicated regions: regions of the genome that were present more often than they should be, and (ii) 8.04 Mbp of falsely collapsed regions: regions with paralogs missing in the reference. Figure 1A describes the issues with these regions that can lead to incorrect mapping of reads and subsequent biases in analysis. This has potential implications across current published work as these regions include medically relevant genes that have been reported through, e.g., GTEx and GWAS studies [17, 18]. To improve mapping and variant calling, we generated a modified GRCh38 reference by first masking out the 1.2 Mbp of extra copies of falsely duplicated regions. From the collapsed regions, we selected a targeted set of three medically relevant genes (*MAP2K3*, *KCNJ18*, and *FANCD2*), two human-specific duplicated genes (*GPRIN2* and *DUSP22*), and their homologous genes and pseudogenes. For the GRCh38 missing genes, we used the T2T-CHM13 reference genome, which was found to correct all identified duplication errors [10]. Specifically, we identified all duplicate homologs in T2T-CHM13 syntenic to the “collapsed” region in GRCh38 and by comparing the references, narrowed in on genomic loci not represented in GRCh38. We then added these missing sequences as decoys to our modified GRCh38 reference genome. See “Methods” for details.

Using this modified reference with masking and additional decoys, we developed a new approach (FixItFelix) [19] to efficiently re-align only the affected reads to the modified GRCh38, correcting existing BAM/CRAM files and subsequent variant calling. FixItFelix is open source (MIT license) and has different modules for short-read, long-read DNA and RNA sequencing reads. Using FixItFelix, a ~30× genome coverage BAM file can be corrected with the new reference in around 4~5 min CPU time, whereas traditional remapping often takes ~24 CPU hours. The left panel of Fig. 1B shows an outline of FixItFelix. To ensure that we captured potential mis-mappings, we collected reads originally mapping to any region homologous to the falsely duplicated and falsely collapsed regions, and remapped them to our modified GRCh38 reference. We also tested FixItFelix with whole genome mapping (the right panel of Fig. 1B), to ensure our novel approach appropriately called variants and we found the results were concordant. Figure 1C shows the location and the number of regions that are impacted by issues along the GRCh38 reference genome. Additional file 2: Table S1 & S2 contains the coordinates of falsely duplicated regions and falsely collapsed regions and the genes that are present in these regions. These genes have problematic coverage in large studies like gnomAD that use GRCh38, with lower than expected coverage for falsely duplicated genes like *CBS* and *KCNE1* (as shown in Fig. 1D), and higher than expected coverage in parts or all of the collapsed genes like *KCNJ18* and *GPRIN2* (as shown in Fig. 1E).

### Improving variant calling with modified GRCh38 based on GIAB

To measure if the modified GRCh38 reference improves mapping quality and variant calling, we first performed a series of experiments on the well-studied dataset of the HG002 sample. For benchmarking, the variant calls using the modified reference genome, the reads were extracted from the original mapping (35× coverage and 2×150 bp Illumina short reads mapped to the original GRCh38 reference) and then remapped to the modified GRCh38 reference sequence using FixItFelix.

**Fig. 2:**
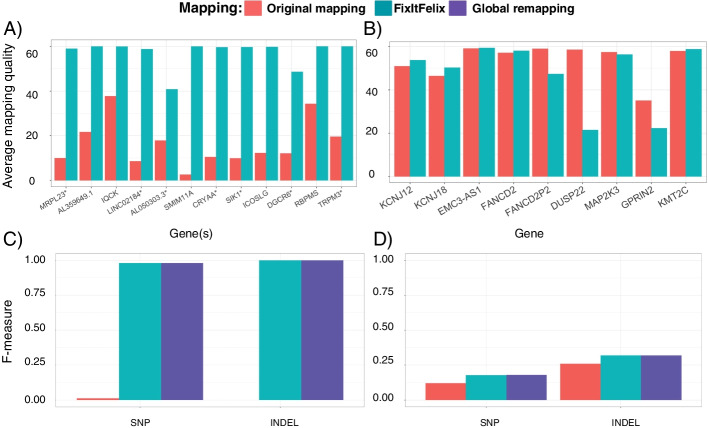
Performance improvements over modified GRCh38 via remapping using FixItFelix. **A** Improvements in mapping quality for duplicated regions. **B** Changes to mapping quality for collapsed regions. **C** SNV and INDEL calling is improved over the mapping to the GRCh38 reference across six genes that are covered in CMRG benchmarking. Furthermore, it clearly shows that a regional approach (mapping to modified reference using FixItFelix) is concordant with global remapping. **D** SNV and INDEL calling improvement for collapsed regions again highlighting that global alignment and regional alignment show similar accuracy

As the falsely duplicated regions were masked in the modified reference sequence, it was expected that the mapping quality (MAPQ) would be improved. This is because the reads that were ambiguously aligned to duplicated regions would be mapped to a single region. In contrast, the mapping quality was expected to be reduced for collapsed regions as the reads could be mapped to two different regions, i.e., the collapsed region and the decoy, instead of the one collapsed region (see Additional file 1: Fig. S1). Our experiments showed that the mapping quality for falsely duplicated regions significantly improved with 78% fewer reads that were mapped to multiple locations (MAPQ = 0: 358,644 in original vs 103,392 in remapping) in the original mapping (Wilcoxon rank sum test *p*-value = 7.396e−07). Figure 2A shows the details per region. Conversely, the mapping quality analysis of collapsed regions showed that the number of read mapping to different locations was increased by 20% (MAPQ = 0: 111,685 reads in original vs 192,280 reads in remapping) as compared to original mapping. Nevertheless, the average mapping quality was as expected, reduced as shown in Fig. 2B. See Additional file 2: Tables S3 & S4 for details.

Given the changes in mapping, we next assessed the variant call performance using the benchmark set for challenging medically relevant genes (CMRG v1.0), which included five medically relevant genes (*KCNE1*, *CBS*, *CRYAA*, *TRAPPC10*, *DNMT3L*) affected by our masking of false duplications and one gene (*KMT2C*) affected by our decoys for collapsed duplications [13]. The GIAB developed this curated benchmark using de novo assembly, which identified and correctly resolved the falsely duplicated regions. For falsely duplicated regions, the short-read BWA MEM-GATK [20] variant calls for both SNV and INDEL greatly improved when the modified reference was used for remapping with FixItFelix (Fig. 2C). For SNVs, the recall score achieved by remapping was 1.0, which was improved significantly compared to the original mapping (0.007). Similarly, the precision and F-measures from the remapping (0.961 and 0.980 respectively) also improved significantly compared to the original mapping (0.063 and 0.012 respectively). For INDEL calls, the remapping was also able to call all 16 true variants, i.e., recall score 1.0 (original mapping: 0.0, no true INDEL calls) with a precision of 1.0 (original: 0.0). See Additional file 2: Table S5 for details. For the falsely collapsed regions, we observed that remapping produced mainly improved precision by removing false positive variants caused by mismapped reads (as shown in Fig. 2D). For SNV calls for *KMT2C* only, the recall improved from 0.949 to 0.974 and the precision score from 0.064 to 0.098. For INDEL calls, remapping did not show any improvements for the *KMT2C * gene as compared to the original mapping. However, there was an improvement in both the precision (0.152 to 0.195) and thus the F-measures (0.259 to 0.319). See Additional file 2: Table S6 for details.

We have measured the outcomes by realigning the reads from the entire genome as well using FixItFelix. Figure 2 C and D show the concordance of the results.

Next, we compared our results by remapping to the T2T-CHM13 (v2.0) reference and then lifting over alignments to the original GRCh38 reference using LevioSAM2 [21]. LevioSAM2 can perform the coordinate conversion between two assemblies, e.g., T2T-CHM13 and GRCh38, using a chain file that contains pair-wise alignment information of two reference sequences. Additional file 2: Table S7 provides detailed performance metrics. For the falsely duplicated regions, FixItFelix performed slightly better than the liftover approach for SNV callings (F-measure: 0.980 vs 0.973) and better for INDEL calling (F-measure: 1.000 vs 0.583), primarily due to increased false negatives with LevioSAM2. However, for the collapsed regions, LevioSAM2 showed a slightly improved performance for both SNV (F-measure: 0.180 vs 0.177) and INDEL callings (F-measure: 0.405 vs 0.319). For the runtime, we saw obvious disadvantages as the first step is to remap the reads to T2T-CHM13 reference sequences before they can be lifted back over to GRCh38. To measure this, we used FixItFelix to remap the reads in the corresponding T2T-CHM13 regions and then only lifted these reads over (see “Methods”). Thus, the overall runtime (both duplicated and collapsed) for LevioSAM2 was around 8~9 min, that is 50% slower than FixItFelix directly.

### General improvements of variant calling

The CMRG benchmark dataset that was used for validation is limited by the number of genes it characterized. Thus, many improvements are not covered. Therefore, expanding on the approach used for CMRG, we utilized the phased HiFi assembly [6] and dipcall [22] (see “Methods”) and treated the resulting VCF and BED as a draft benchmark. Furthermore, we confirmed that GIAB CMRG and dipcall results were concordant across all regions included in CMRG (see Additional file 2: Table S8).

**Fig. 3:**
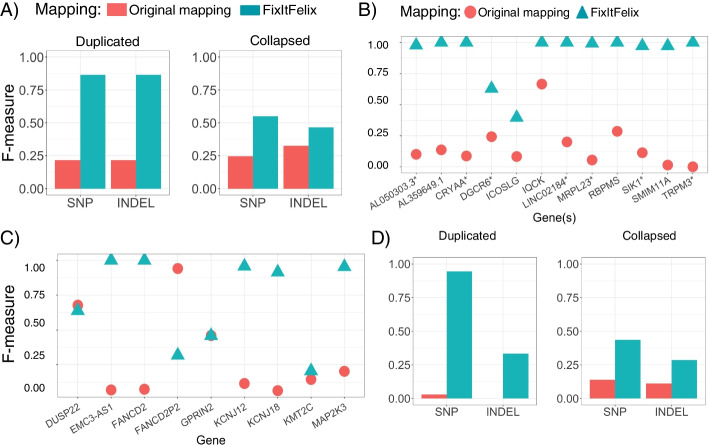
SNV benchmarking on HG002 using dipcall benchmark set. **A** Benchmarking of SNV and INDEL using GATK variant calls with original mapping (original GRCh38 reference genome) and FixItFelix (remapping with modified GRCh38 reference). The region-wise (labeled by gene(s) inside the region) benchmarking is shown for **B** duplicated regions, where more than one gene was impacted at some regions and **C** collapsed regions, where each region contains only one gene. **D** Benchmarking of whole-exome sequencing datasets that show the improvement of variant calling on exon regions when the modified reference was used

The SNV and INDEL calls were clearly improved by the modified reference in falsely duplicated regions when benchmarking against the draft benchmark, i.e., dipcall VCF and BED (as shown in Fig. 3A). For SNVs, the improvement of variant calls is significant (Friedman rank sum test *p*-value = 0.02307) as the recall went up from 0.13 to 0.85 with the improved precision score. We also observed similar performance for INDEL calls. For collapsed regions, the precision and thus overall F-scores were improved from 0.22 and 0.32 to 0.452 and 0.453 respectively. Thus, we showed that across more genes, not covered in CMRG dataset, the proposed reference modifications are showing an overall improvement for mapping and variant calling.

To determine if variant accuracy differs between affected regions, we analyzed the variant call performance for each individual region impacted by falsely duplicated and collapsed events. For falsely duplicated regions, we observed a clear improvement of SNV calls by using modified references over the original. The F-measures were 1.0 for six out of 12 regions and 0.97~0.99 for four other regions (see Fig. 3B). The recall, precision, and F-measure scores for all regions are shown in Additional file 2: Tables S9 & S10. The two regions with lower performance had additional true segmental duplications that caused mapping challenges even after masking the falsely duplicated sequence. Collapsed duplication showed more variable performance between regions. The modified reference substantially improved accuracy in the five regions containing genes *FANCD2, EMC3-AS1, KCNJ12*, *KCNJ18*, and *MAP2K3*. The *KMT2C* gene showed moderate improvements, but some sequences appeared to still be missing from GRCh38. However, two genes (*DUSP22* and *GPRIN2*) showed slightly lower F-measures and for pseudogene *FANCDP2*, it went down from 0.941 to 0.318. Upon curating alignments, the larger regions containing *FANCD2*, *DUSP22*, and *GPRIN2* appeared to have challenges both in making reliable assembly-based benchmarks and in mapping reads to GRCh38 with the decoys. GRCh38 may have structural errors in these genes in addition to the collapsed duplication, and common structural variation in the population may impact variant call accuracy. While most parts of the *FANCD2*, *DUSP22*, and *GPRIN2* genes affected by these decoys appear to be improved, these regions are more complicated and some regions in and around these genes may have performance reduced by the decoys.

We also compared the variant calling performance for both falsely duplicated and collapsed regions with the original and modified GRCh38 reference genome using long-read sequencing (PacBio HiFi and ONT) of HG002 sample (see “Methods”). Using the PacBio HiFi reads, we observed a slightly lower precision but with an overall improved F-score for SNV (from 0.251 to 0.308) and INDEL (0.421 to 0.452) calling as well for duplicated regions. We also obtained an improved precision and recall and thus overall F-score for SNV (0.242 to 0.327) and INDEL (0.451 to 0.564) calling across the collapsed regions. Similarly, for ONT reads we also observed a general improvement in SNV and INDEL calls. Additional file 2: Table S11 contains all the results for long-read datasets.

Given the clear improvement across whole genome sequencing (long and short reads) data in most regions, we next investigated the effects of the modified reference to whole exome sequencing (WES) and RNA sequencing. For WES-based SNV calls in duplicated regions, the use of the modified reference greatly improves the performance with a high recall score of 0.909 with a precision 0.984 and F-measure 0.945 as compared to the original reference-based recall, precision, and F-measures 0.015, 0.333, and 0.029, respectively (as shown in Fig. 3D). For SNV calls in collapsed regions, we observed a slightly increased recall (0.389 vs 0.333) with the modified reference and higher precision (0.632 vs 0.067). So, the F-measure improved from 0.159 to 0.472 (as shown in Fig. 3D). There were only a few INDEL in the erroneous regions (nine in duplicated and three in collapsed regions) found in the dipcall benchmark set. For both regions, better precision and F-measure were observed when GATK calls were made using the modified reference (as shown in Fig. 3D). The detailed results from the evaluation tool are given in Additional file 2: Table S12.

Next, we assessed the performance improvement on RNA sequencing data for the HG002 B-Lymphocyte cell type. Here we used STAR [23] aligner within FixItFelix and subsequently GATK for variant calling. First, we assessed the coverage/expression changes across the genes (see Additional file 2: Table S13 for details). For falsely duplicated regions, we observed a higher coverage (2.22× more coverage on an average) for all exon regions when the modified reference was used. For falsely collapsed regions, we did not observe reads across the genes *FANCD2*, *KCNJ12*, and *KCNJ18*, i.e., maybe the genes are not expressed. Only regions around *DUSP22* and *MAP2K3* showed mapping reads. As expected, the decoys reduced the coverage over the initially collapsed regions, whereas for the remaining regions, the coverages were exactly the same for both references.

The evaluations of variant calling were performed by comparing GATK variant calls to the draft benchmark dataset (generated by running dipcall with modified GRCh38 reference). For duplicated regions, the RNA-seq F-measure increased from 0.148 (with original reference) to 0.634 when the modified reference was used. We observed a significant increase in recall (0.08 to 0.52) in those duplicated regions but also a slight decrease in precision (from 1.0 to 0.81). Across the collapsed regions, we only were able to assess six SNVs which showed the same performance for both the references (recall: 0.5 and precision: 1.0). See Additional file 2: Table S13 for details.

### The modified reference improves variant detection across ancestries

**Fig. 4:**
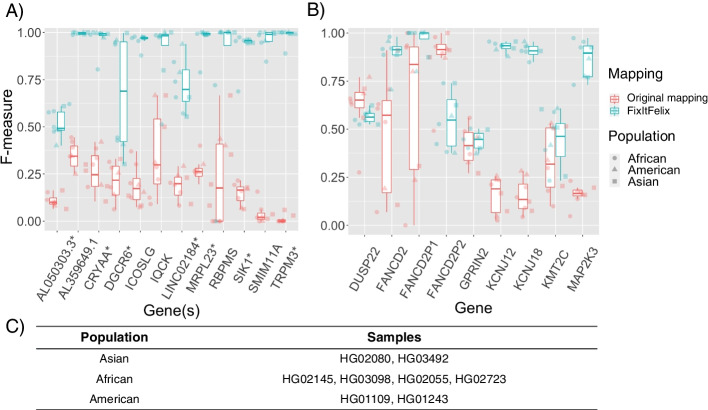
F-measures benchmarking for eight pan-genome samples. SNV benchmarking of both **A** falsely duplicated and **B** falsely collapsed regions using original and modified GRCh38 references. The *x*-axis is labeled with gene names that are impacted due to false duplication/collapsed errors. For some of the duplicated regions, more than one gene are impacted and gene names with * (star) subscripts represent several other genes including the labeled gene name. The red and green box plots correspond to the original and modified GRCh38 references respectively. **C** The eight pan-genome samples chosen from 1KGP consist of African, American, and Asian samples

So far, we have shown a clear improvement of the variant calling for HG002, a sample with European ancestry. Nevertheless, to ensure these results apply beyond the European HG002, we extended our benchmark using dipcall to eight additional individuals from the T2T Diversity Panel (see “Methods”). We first generated the draft benchmark sets for these samples by calling variants with dipcall that uses hifiasm assemblies of their paternal and maternal haplotypes. The Illumina data was then processed similarly than before and we compared the GATK variant performance before and after applying FixItFelix (see “Methods”). This collection includes datasets for four African (HG03098, HG02055, HG02723, and HG02145), two American (HG01109 and HG01243), and two Asian (HG02080 and HG03492) samples. For the four African samples, we observed a significant improvement of F-measure for all false-duplication regions. We also observed similar improvements for the two American and two Asian samples. The whisker plots of Fig. 4A show the F-measures for all eight samples. Overall, the F-measures are significantly higher for all samples (Kolmogorov-Smirnov test *p*-value < 2.2e−16) and all duplicated regions when the modified reference is used. The regions covering the genes *CRYAA/CBS/U2AF1*, *ICOSLG*, *SIK1*, *SMIM11A/KCNE1*, and *TRPM3* show nearly perfect F-measure of 1.0 for most of the samples. The F-measure comparison for SNVs of all regions of individual samples is shown in Additional file 2: Table S14.

We observed a decrease in recall scores for the collapsed regions that contain *DUSP22* gene and *FANCD2P2* pseudogene. This could be due to the fact that some reads that had correctly aligned to these regions are now aligning to the corresponding decoy sequences or have too low mapping quality. However, the variant precision improved, showing the modified reference’s ability to exclude false variants that were initially called when the original GRCh38 reference was used. The above pattern was observed for all African, American, and Asian samples. The collapsed regions that contain genes such as *FANCD2*, *KCNJ12*, *KCNJ18*, and *MAP2K3* showed a significant improvement over F-measures (Wilcoxon rank sum test: *FANCD2 p*-value = 0.003824, *KCNJ12 p*-value = 0.0001554, *KCNJ18 p*-value = 0.0001554 and *MAP2K3 p*-value = 0.0001554, Fig. 4B). This was consistent among all eight samples that we used in our analysis. For half of the regions, the improvement was significant with an 80% increase in F-measures when the modified reference was used. Additional file 2: Table S15 contains the F-measure comparison of all regions for individual samples. Thus, the improved accuracy across different samples and different populations demonstrates that the errors are not specific to the HG002 sample.

### New realignment allows scaling to thousands of human genomes

Given the improvements across the whole genomes for multiple ancestries, it is clear that FixItFelix, together with the modified version of GRCh38, improves variant calling and mapping in multiple important regions of the human genome. We next applied FixItFelix across 4174 publicly available samples to measure the benefits of joint calling and obtain more insights into these genes. These samples include high coverage (target depth of 30x) sequencing of 3199 individuals from the 1KGP [16], 828 individuals from the Human Genome Diversity Project (HGDP) [24], and 147 quality control sequences from the Trans-Omics for Precision Medicine program (TOPMed) [25].

For mappings to both the original and modified reference, we calculated the mean mapping quality within each of the falsely duplicated and collapsed regions for each of the 4174 samples. Mapping qualities for the falsely duplicated regions were consistently improved by using the modified reference (see Additional file 1: Fig. S2). For the collapsed regions, the mapping quality either decreased or remained roughly the same when using the modified reference (see Additional file 1: Fig. S3). These results are consistent with the evaluations of mapping quality described in previous sections.

**Fig. 5:**
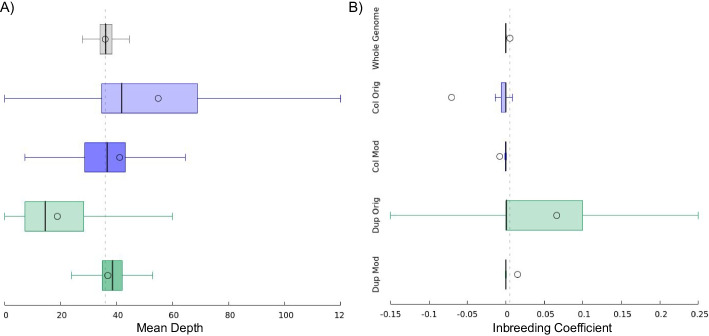
Mean read depth and inbreeding coefficient distributions. Distributions of **A** mean read depth and **B** inbreeding coefficient of variants for the whole genome (excluding duplicated and collapsed regions), collapsed regions using the original reference, collapsed regions using the modified reference, duplicated regions using the original reference, and duplicated regions using the modified reference. Distribution means are indicated with circles, whiskers denote min/max within ± 1.5 times the interquartile range, and box lines denote quartile boundaries

Since falsely duplicated sequences would have mappings spread across two sequences (Fig. 1A), it is expected that the read depths for these regions would be decreased. Likewise, reads from two different sequences would be mapped onto the same collapsed sequence, resulting in an increase in read depth for such regions. In order to assess whether this was happening in the falsely duplicated and collapsed regions that we identified, we calculated the mean depth for each variant within both call sets and compared the affected regions to the rest of the genome (see Fig. 5A). With the original reference, the mean read depth for the collapsed regions (51.5) was approximately 1.5 times higher than the mean read depth of the unaffected regions (33.7). Similarly, the mean read depth for the falsely duplicated regions (17.7) was nearly half that of the unaffected regions. With the modified reference, these deviations in mean read depths for the collapsed and duplicated regions receded to 38.4 and 34.5 respectively.

These types of mapping errors can often lead to artificial departure from Hardy-Weinberg equilibrium [26], and we saw this in our experiment. In variant calls generated from the original mapping, we observed elevated rates of variants with heterozygous deficiency (indicated by positive inbreeding coefficients) within the falsely duplicated regions, likely caused by read mapping to the false copy of the sequence when they have a variant that matches the false copy, which also results in lower coverage. Conversely, we observed excessive heterozygosity (indicated by negative inbreeding coefficients) within the collapsed regions, likely caused by paralogous sequence variants (PSVs) in read mismapping to each region from the missing paralogous region [11], also resulting in higher coverage (see Fig. 5B and Additional file 1: Fig. S4). These departures from Hardy-Weinberg equilibrium were improved for both the falsely duplicated and collapsed regions when using the modified reference.

### GRCh38 errors impact gene expression quantification and lead to artifactual cis-eQTLs

**Fig. 6:**
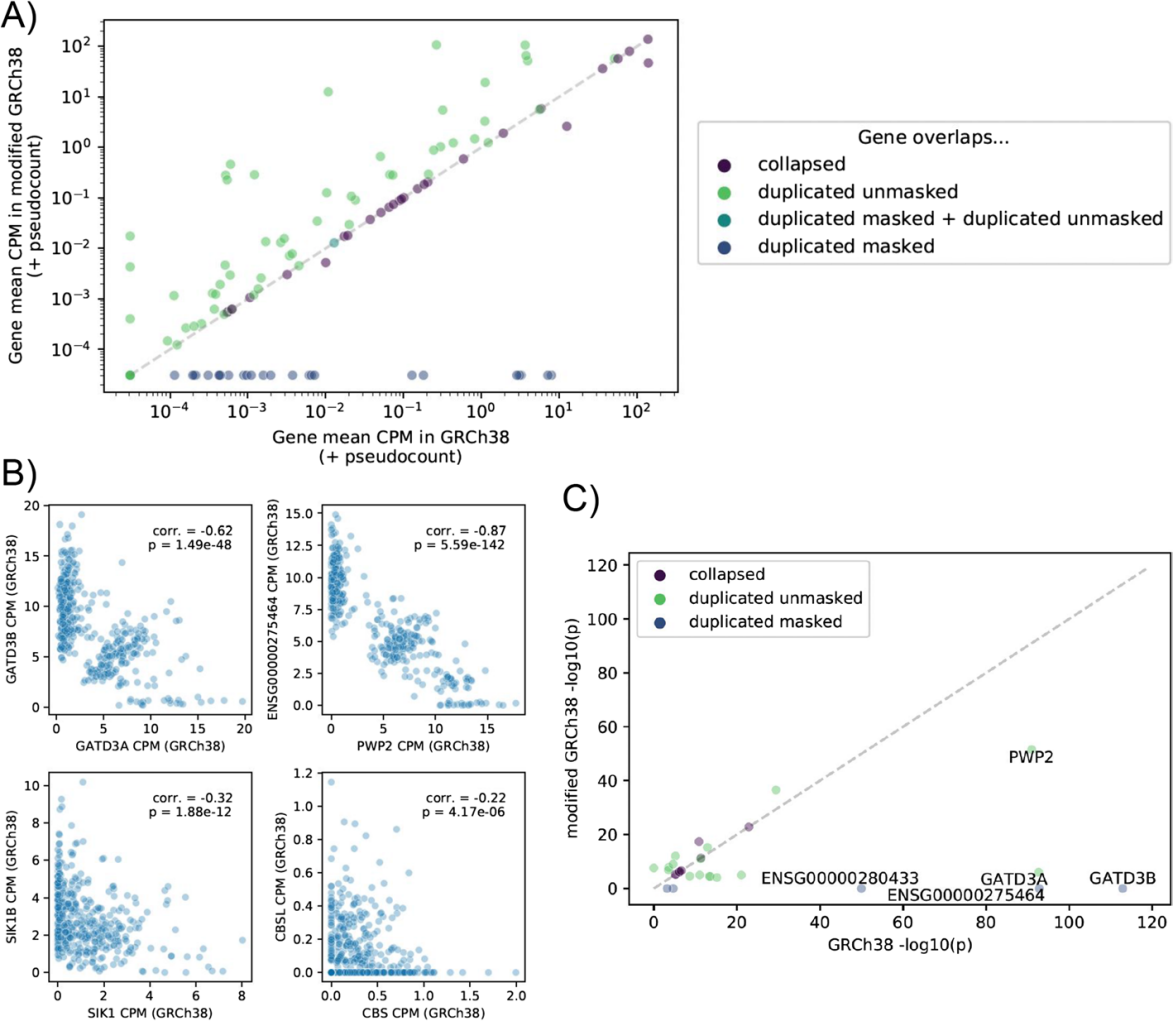
RNA-Seq eQTL analysis. **A** Mean counts per million read pairs (CPM) across RNA-seq samples for each gene overlapping collapsed, duplicated masked, or duplicated unmasked regions, using GRCh38 or the modified GRCh38. **B** Per-sample CPM of a true gene (*x*-axis) and its false paralog (*y*-axis) for four selected pairs, displaying a significant negative correlation between paralogs. **C** Comparison of cis-eQTL *p*-values for genetic variants most significantly associated with each gene’s expression in GRCh38 or the modified GRCh38. Only genes that are an eGenes in at least one of the references and that are within collapsed, duplicated masked, or duplicated unmasked regions are shown; if a gene was not included in the eQTL scan for one of the references (because the expression was too low), the *p*-value was set to 1 for the purposes of this panel. The 5 plotted genes showing the most extreme change in top cis-eQTL *p*-value between the references are labeled

We hypothesized that errors in the original GRCh38 reference impact analyses beyond variant calling. To explore this further, we mapped 449 1KGP lymphoblastoid cell line RNA-seq datasets [27] (see Additional file 2: Table S16) to the modified and the original reference, and compared gene read counts between the references (Fig. 6A). As expected, when using the modified reference, the number of read mapping to (unmasked) duplicate genes generally increased. These reads are commonly multi-mapped or mapped to the false paralog in the original reference (Additional file 1: Fig. S5); frequently, the expression of the two paralogs is negatively correlated (Fig. 6B). This suggests that in the case of falsely duplicated regions, the false paralogs may compete with the true gene for RNA-seq reads during mapping. Changes within collapsed regions were more restricted, with only two neighboring genes (*DUSP22* and *ENSG00000287265*) in collapsed regions showing both substantial changes in gene counts and mean counts per million reads (CPM) > 0.1 in either reference. As the expression of these genes may vary across tissues, the changes observed for any one gene may vary as well.

To demonstrate the impact of these changes on downstream analyses, we used gene counts from the modified and original reference, along with the corresponding genotypes, to perform a *cis*-expression quantitative trait locus (*cis*-eQTL) analysis using these samples. We identified 10,450 genes with at least one significant eQTL signal (eGenes; 5% FDR) in at least one of the references (10,437 eGenes in GRCh38; 10,417 eGenes in modified GRCh38). Five genes in duplicated masked regions (*ENSG00000275464*, *ENSG00000277117*, *ENSG00000280433*, *GATD3B*, *SIK1B*) were significant eGenes in GRCh38. We compared the *p*-values for the variants most significantly associated with each gene’s expression in either reference (Fig. 6C; Additional file 1: Fig. S6; Additional file 2: Table S17). As expected, genes in unaffected regions showed little change between the references. Across all genes that were an eGene in at least one of the references, 22 genes showed a change in nominal *p*-value (for association with the most strongly associated variant) between the references exceeding 1 orders of magnitude, with only one of these (*TRAPPC10*) located outside of affected regions.

Several eQTL signals in the original reference weakened considerably in the modified reference. The *PWP2* and *GATD3A* GRCh38 cis-eQTL signals represent particularly striking examples. To explore the reason for this change, we examined the top variant–gene expression associations for GRCh38 eGenes showing a weaker eQTL nominal *p*-value in the modified reference than in GRCh38 (at least one order of magnitude smaller). For each of these genes, the RNA-seq sample genotypes of the top associated GRCh38 variant (eVariant) were always identical between the references (excluding genes masked and therefore untested in the modified GRCh38), suggesting that the decrease in eQTL signal strength was due to changes in gene expression quantification rather than genotype calling. Interestingly, for these genes, we found that the change in gene read counts between the two references were often clearly associated with individuals’ genotypes, suggesting that the ability to map a read to the gene in the original reference was dependent upon genotype and the original eQTL signal was likely artifactual (Additional file 1: Fig. S7 & S8). Consistent with the hypothesis that paralogs between the duplicated regions compete for RNA-seq reads in the original reference and that a read preference for one or the other is correlated with genotype, the eVariants for the strong signals that weakened in the modified reference frequently correlated with the expression of the (often distant) paralogs, with the opposite direction of effect (Additional file 1: Fig. S9). This hypothesis is also supported by the higher than expected fraction of homozygous variants in false duplications in the Hardy-Weinberg equilibrium analysis above. We, therefore, urge caution when interpreting gene expression analysis results for genes in affected GRCh38 results; for example, the top variants associated with *PWP2* and *GATD3A* gene expression in GRCh38 in our analysis (associations that weaken considerably when running the analysis in modified GRCh38) are also the top eVariants for these genes in one or more GTEx tissues [17].

## Discussion

The errors due to falsely duplicated and collapsed events in the GRCh38 reference (GRCh38.p13) have greatly impacted the variant calls across 9.24 Mbp including 33 protein-coding genes of which 12 are highly medically relevant genes (Fig. 1). Therefore, previous studies on these impacted genes using GRCh38 contain erroneous information across variant calls or even expression analysis was used for any subsequent analysis (e.g., gnomAD, GTEx) (e.g., Fig. 1D). In this work, we focused on fixing these erroneous regions in GRCh38 (p13 release) reference by masking the extra copy of duplicated regions and adding newly identified decoys for falsely collapsed regions, thus improving among the recent release GRCh38.p14. To circumvent the need to remap to the entire genome or even larger cohorts, we developed an efficient methodology FixItFelix to correct existing bam files with minutes of computing, thus allowing for a rapid improvement across these regions and genes. We show that the modified reference improves SNV calling for long and short reads of the whole genome and whole exome data, as well as for RNA-seq data. FixItFelix further can be applied also for future corrections as it is not implemented specifically for the GRCh38 genome. We showed this by using FixItFelix to also benchmark an alignment liftover approach, where FixItFelix aligned reads to the T2T-CHM13 reference directly. Overall, these benchmarks further supported the fact that FixItFelix is the fastest way to improve variant calling for these important genes. Furthermore, it is easy to use FixItFelix also for other genome model or non-model organisms to adjust existing collections of BAM/CRAM. This only requires adjustment of the bed files that FixItFelix utilizes for the remapping.

Using a recently published study [13], we identified multiple genes that were accidentally duplicated leading to a low mapping quality and thus missing variants along 12 medically relevant genes and also other regions of the genome. Furthermore, we collaborated with the T2T consortium to identify multiple genes that were collapsed, enabling the identification of multiple haplotypes across genes in these regions [11]. In this study, we first assessed if these biases are impacting multiple ethnicities and sequencing assays (WES, WGS, RNA-seq) showing profound implications on current genomic resources and public databases. Second, we provide a solution to these issues by introducing novel decoys and modifications to the GRCh38 reference together with FixItFelix to correct an existing BAM or CRAM file within minutes. For whole genome sequencing, we show that our approach improves variant calls across all falsely duplicated regions and that it clearly improves some collapsed regions but others are more complex. We provide a new GRCh38 reference fasta that masks the extra copy of all false duplications and includes new decoys associated with the genes *KCNJ12*, *KCNJ18*, *KMT2C*, and *MAP2K3*. We provide an additional fasta with decoys for *FANCD2*, *EMC2*, *FANCD2P1*, *GPRIN2*, and *DUSP22* (see “Availability of data and materials”), which we found reduces false positives but can also substantially increase false negatives in *FANCD2P1*, *GPRIN2*, and *DUSP22* due to reduced mapping quality and possible structural errors in GRCh38 or structural variation. Other limitation of FixItFelix is that the program itself focuses on these regional approaches rather than a genome-wide approach. This also includes that these regions need to be identified first based on genomic alignments and this might require some expertise. Thus applying it to other genome builds will be possible, but requires some steps before hand.

For RNA sequencing, the evaluation remains challenging as the genes and therefore the variants that we utilized to identify true or false positives are not always expressed. Nevertheless, the coverage overall has shown benefits. Furthermore, we show that a subset of falsely duplicated genes and genes in collapsed regions show substantial changes in estimated expression when quantifying expression with GRCh38 or the modified GRCh38, and cis-eQTL results uncovered evidence of genotype-associated mapping differences between the references that may lead to false eQTL signals when using the original GRCh38. We urge caution when interpreting the results of GRCh38-based gene expression related analyses for genes overlapping these duplicated or collapsed regions.

Ongoing efforts from T2T and other consortia are producing genomes that resolve these regions, e.g., T2T-CHM13. While we utilize this information to create the decoys that we introduced, we are also aware of all the annotation resources that are built around GRCh38 (e.g., gnomAD) to rank variants for certain phenotypes. Furthermore, millions of exons and genome sequencing datasets have been or are currently analyzed on GRCh38 (All of Us, TOPMed, etc.). Thus, the re-analysis by using T2T-CHM13 on these large consortia data may not be an efficient solution. Therefore, to overcome the computational burden for large consortia, our solution is ideal as it only takes a few minutes of additional analyses to improve and correct the mapping of reads in these medically relevant regions that are either collapsed or duplicated by chance. In addition, with the creation and improvements of human assemblies, the likelihood is high that we might identify further issues with the GRCh references. Here, FixItFelix can be quickly adapted to include ancestry-specific sequences or true errors in GRCh38 going forward.

## Conclusions

This study highlights the issues with GRCh38 and shows their impacts across multiple ancestries for different genomic assays. Furthermore, we provide an efficient solution that can be applied by every cohort or study. We provide a modified GRCh38 reference that corrects both falsely duplicated and collapsed errors that impacted 33 protein-coding genes including 12 medically relevant genes such as *KCNE1*, *CBS*, and *MAP2K3*. Using this modified GRCh38 reference together with our tool improves variant calling in these challenging genes across short and long reads. Furthermore, it corrects previous errors that are published in public variant annotation databases and provides unbiased insights into eQTL studies. Thus, together it forms a significant improvement that human genome studies need to include for their analysis.

## Methods

### Collapsed and duplicated regions

We created reference decoys for ten genic regions with evidence of GRCh38 collapsed duplications (*TAS2R46*, *MAP2K3*, *KCNJ18*, *KATNAL2*, *FANCD2*, *LPA*, *MUC3A*, *KMT2C*, *GPRIN2*, and *DUSP22*). First, we identified syntenic regions in T2T-CHM13 (Additional file 2: Table S18) by BLAT [28] comparisons and matching gene annotations. Next, using T2T-CHM13 annotated segmental duplications [29], we identified all homologs, extracted their sequences and gene annotations (UCSC Genome Browser Table Browser; T2T-CHM13 v1.0), and compared them to GRCh38 using a combination of BLAT and minimap2 [30]. Finally, syntenic duplicate regions between references were flagged as those sharing the same gene annotations and highest sequence similarity via manual curation. T2T-CHM13 sequences of duplicate regions not represented in GRCh38 were included as decoys. Further, all read mapping to GRCh38 syntenic duplicate regions were extracted from original BAMs and remapped to the new modified GRCh38 reference. We chose not to include *TAS2R46*, *KATNAL2*, *LPA*, and *MUC3A* because they did not have simple decoy sequences that could be added.

### Remapping tool—FixItFelix

Our remapping tool, FixItFelix, extracts only the mappings of the regions of interest from the existing whole genome mapping BAM/CRAM file and then extracts sequences for those regions (duplicated or collapsed) and finally realigns the sequences to the modified GRCh38 reference. This is significantly faster (4~5 min) than global whole genome mapping (may take >24 h). Following are the detailed steps of FixItFelix (see Fig. 1B). First, the regions in the original BAM/CRAM file, i.e., mapped to GRCh38 reference genome, corresponding to input BED regions (duplicated or collapsed) are extracted using samtools [31] (v1.12) with “-F 2316” flag (primary alignments, not supplementary alignments, reads are not unmapped, and mate pairs are not unmapped). The extracted alignments were further filtered to make sure that we are keeping the alignments only for paired-end reads with unique read names. Then, the sequences are extracted from these alignments using the “samtools fastq” command. Finally, the extracted reads were mapped to the modified reference genome using BWA [32] (v0.7.15).

### Short-read mapping genome wide

For comparing the mapping quality, we first aligned the reads to entire reference genomes (original and modified GRCh38). The short reads were mapped to both original and modified reference using the bwa-mem algorithm of BWA (v0.7.15) aligner tool with minimum seed length (-K) set to 100,000,000 and all other parameters were set to its default value. The mapping qualities were evaluated using a customized script that used the samtools (v1.12). We also manually examined a few of the mapping regions of our interest using the Integrative Genomic Viewer (IGV) tool (v2.12.3).

bwa mem -t 8 -R @RG\tID:0\tSM:HG002\tLB:HG002\tPU:HG002_38_nodecoy\tCN:BCM\tDT:2021-03-10T00:00:00-0600\tPL:Illumina GCA_000001405.15_GRCh38_no_alt_analysis_set.fasta HG002.novaseq.pcr-free.35x.R1.fastq.gz HG002.novaseq.pcr-free.35x.R2.fastq.gz

### Variant calling

To evaluate the variant calls when mapped to the original and modified reference, we used both genome-wide mapping and the mappings corresponding to regions impacted by duplicated and collapsed errors. We use our remapping script to extract BAM regions from both the original mapping and the mapping to the modified reference genome.

Single-sample variant calling for the whole genome, regional and remapped regions was done using GATK (v3.6) Haplotypecaller [33]. Jointly genotyped call sets for 1KGP, HGDP, and TOPMed samples were generated with the TOPMed variant calling pipeline. The customized scripts for variant calling are available at our Github repository (see “Code availability”).

### Long-read variant calling

We called variants (SNVs and indels) using existing long-read aligned bam file and PRINCESS (v1.0) [34], with the “snv” option and default parameters. The “snv” option from PRINCESS calls implicitly Clair3 [35] (v3.0.1.11); for the specific regions of interest (used the `--bed_fn` option to specify the bed file).

### Evaluating variant calls

Our evaluation for the HG002 dataset was done using two different benchmark sets: (A) GIAB challenging medically relevant gene (CMRG) benchmark set and (B) dipcall benchmark set. The dipcall benchmark was chosen as CMRG covers only a small set of regions. For 1KGP samples, the HG002 WES dataset, and HG002 RNA-Seq dataset, we used only the dipcall benchmark set.

Using GIAB CMRG (v1.0) dataset, the benchmarking of variant calls was performed using the hap.py [36] tool (v0.3.14) that used the high-confidence region BED file of CMRG set (-f parameter) and duplicated/collapsed BED regions (-T parameter). The reference (-r parameter) was appropriately chosen for original mapping and mapping to the modified reference.

For the eight 1KGP samples, we first run dipcall (v0.3) by taking their publicly available maternal and paternal assemblies (hifiasm tool and PacBio HIFI reads were used for assemblies). The dipcall was run with the modified reference genome and the assemblies, the output VCF file, and BED file were used as benchmark set and high-confidence regions for evaluation. We again used the hap.py tool for comparing the GATK VCF file with the benchmark VCF file. Our analysis on 1KGP was performed by using the dipcall benchmark set that was generated using the modified reference genome.

For WES and RNA-Seq experiments, we used HG002 dipcall results with the modified reference as the benchmark set. The bedtools (bedtools intersect) were used to extract the exon regions that overlap with duplicated/collapsed regions. The comparison of VCF files containing GATK variant calls and the truth set (dipcall benchmark) was done using the hap.py tool. For RNA-Seq, we followed a specific pipeline of GATK that was designed for RNA-Seq experiments which were different from genome-wide variant calls.

The read depth and Hardy-Weinberg Equilibrium metrics used when evaluating variants in the jointly genotyped call sets were taken from VCF INFO fields that were generated with the TOPMed variant calling pipeline. Specifically, the average read depths and inbreeding coefficients for each variant were taken from the INFO fields respectively named “AVGDP” and “FIBC_P” [25].

### Statistical testing of improvement of mapping quality and evaluated variants

We evaluated the mapping quality of improvement of FixItFelix for both duplicated and collapsed regions using the data from Additional file 2: Tables S3 and S4 respectively. We compared the mapping quality of both strategies using a Wilcoxon rank sum test (in R).

> wilcox.test(original.mapping.quality, fixItFelix.mapping.quality, alternative="two.sided")

The scores of the SNV and INDEL calls benchmark were compared using a Friedman rank sum test (in R) where we compared the F-scores by the mapping strategy (Original, Global remapping, FixItFelix) and type of event (SNP, or INDEL) for both the collapsed and masked regions (Additional file 2: Table S19).

> friedman.test(F1.score ~ Mapping | Type ,grch38_f1scores_supp_table_S19)

Next, we compared the F-score across 12 and nine genes for duplicated and collapsed regions respectively in eight individuals from three distinct ancestries (two from Asian, four from African, and two from American ancestry). For both duplicated and collapsed regions, we aggregated the F-scores for each mapping strategy (Original and FixItFelix) and performed a Kolmogorov-Smirnov test. Finally, for the case of collapsed regions, we analyzed four genes (FANCD2, KCNJ12, KCNJ18, and MAP2K3). The results looked very promising and we also performed a Wilcoxon test (in R) to compare the F-scores of each gene.

> wilcox.test(gene.original.Fscore, gene.FixItFelix.Fscore, alternative="two.sided")

### Comparison to Leviosam2 alignment liftover

We first used FixItFelix to extract the mapping of erroneous regions from the original BAM file and remapping to T2T-CHM12 (v2.0) reference genome using BWA (v0.7.15). Then, we used LevioSAM2 (v0.2.2) and the provided chain file to liftover the T2T mappings to the GRCh38 reference. The output BAM file with file extension “-final.bam” was used for subsequent analysis. However, we observed that several reads were not assigned the read groups. So, we run Picard (v2.6.0) with “AddOrReplaceReadGroups” to assign all reads in the BAM the original read groups.

### Gene expression quantification

Raw RNA-seq reads were mapped to either reference using STAR [23] (v. 2.6.1d; parameters --outFilterMultimapNmax 20 --alignSJoverhangMin 8 --alignSJDBoverhangMin 1 --outFilterMismatchNmax 999 --outFilterMismatchNoverLmax 0.1 --alignIntronMin 20 --alignIntronMax 1000000 --alignMatesGapMax 1000000 --outFilterType BySJout --outFilterScoreMinOverLread 0.33 --outFilterMatchNminOverLread 0.33 --limitSjdbInsertNsj 1200000 --outSAMstrandField intronMotif --outFilterIntronMotifs None --alignSoftClipAtReferenceEnds Yes --quantMode TranscriptomeSAM GeneCounts --outSAMtype BAM Unsorted --outSAMunmapped Within --chimSegmentMin 15 --chimJunctionOverhangMin 15 --chimOutType Junctions WithinBAM SoftClip --chimMainSegmentMultNmax 1). STAR indices were produced using the GENCODE v. 39 GTF file [37] (which was used for all gene expression quantification and eQTL analyses) with option --sjdbOverhang 100. Gene counts were quantified using RNASeQC [38] (v2.3.4; options --stranded rf; for use with RNASeQC the GTF was collapsed using the GTEx [17] script (Github commit 9c6a1c38b)).

To compare read feature assignments for affected genes (used for Additional file 2: Fig. S5), we extracted reads that overlapped affected genes or affected regions in either reference (samtools view -F 2048 -F 256 -L affected_regions_and_genes.bed) and used htseq-count [39] (v0.12.3) to assign them to features (--stranded=yes --type=gene -a 0 --samout=out.sam), extracting feature assignments from the XF tag in the output sam file.

### cis-eQTL analysis

We used sex, four genotype principal components (PCs), and 30 gene expression PCs as covariates for the cis-eQTL analysis. The number of gene expression PCs to include as covariates were determined by running the cis-eQTL scans using anywhere between 0 and 100 PCs (in steps of 5) and selecting the point at which the number of eGenes discovered began to saturate (see Additional file 1: Fig. S10).

Gene expression values used in the cis-eQTL scan were pre-processed as follows:

1. Gene counts were filtered to include only autosomal and chrX genes
2. Genes counts were normalized using the edgeR [40] Trimmed Mean of M-values (TMM) procedure, i.e., computes normalization factors that represent sample-specific biases, as implemented in pyqtl (v0.1.8) function edger_cpm.
3. Lowly expressed genes, defined as those where <20% of samples have a transcript per million (TPM) value of > 0.1, were dropped.
4. TMM-normalized gene expression values were inverse normal transformed.

To generate gene expression PCs to be used as covariates in the cis-eQTL scans, we performed PC analysis (PCA) on the inverse normal transformed gene expression matrix.

Genotype PCA was performed using genotypes in unaffected regions, such that the PCs for the GRCh38 and modified GRCh38 eQTL scans were identical. Genotypes for PCA were generated by filtering to common (MAF ≥ 1%) autosomal SNPs, followed by LD pruning using plink (v. 1.90b; --indep-pairwise 200 100 0.1). EIGENSOFT [41, 42] (git commit 09ed563f) was used for the PCA, computing the top 15 PCs (smartpca.perl with options -k 15 -m 0).

cis-eQTL scans were performed using tensorQTL [43] (slightly modified from v1.0.6; mode = cis, with *q*-value lambda = 0, seed = 2021 and otherwise default parameters), testing variants within 1 Mb of the gene TSS and with in-sample MAF ≥ 1%.

## Supplementary Information

Additional file 1. Supplementary figures; this file contains all the supplementary figures.

Additional file 2. Supplementary Tables; this file contains all the supplementary tables.

Additional file 3. Review History; this file contains the review history.

## Data Availability

Challenging medically relevant regions (CMRG) benchmark for HG002 sample (GRCh38) with high-confidence regions [44] GRCh38 original reference [45] GRCh38 modified reference (used for all analysis in this study) [46] 2nd version of Modified GRCh38 reference that excludes decoys related to *FANCD2*, *DUSP22* and *GPRIN2* genes [47] HG002 HiFiasm assembly used for dipcall [48] Eight HPRC samples [49] Whole Exome Sequencing (hiseq4000,wes_agilent,50x,HG002,grch38) [50] WES high-confidence BED regions [51] HG002 RNA-Seq data [52] HG002 RNA-Seq coding regions BED [53] Code availability FixItFelix [19, 54] Scripts used for plots [55] TOPMed variant calling pipeline [56] Pyqtl [57] GTEx script [58] Modified tensorQTL [59]
