# Supplementary material for: FixItFelix: improving genomic analysis by fixing reference errors
Source: Genome Biol. 2023 Feb 21;24:31. doi: 10.1186/s13059-023-02863-7 (PMC9942314; doi:10.1186/s13059-023-02863-7)
Supplement: Supplementary file 3 — Additional file 3. Review History; this file contains the review history. [file 13059_2023_2863_MOESM3_ESM.docx]

**1^st^ round**

**Reviewer 1**

Fixing errors in the reference assembly are an unfashionable yet very important part of genomics, as so much downstream analysis in medical genomics relies on, and will continue to rely on, mapping sequencing reads to a reference sequence and interpreting differences as genuine variation in the diploid genome of the individual sequenced. This paper is very welcome, as it addresses particular erroneous regions, provides an accessible software tool to fix existing sequence alignments, and demonstrates the importance of this by illustrating variant calling on medically-important genes in the fixed region.
Overall, this is an excellent paper. Clear to read, interesting, thorough and important. I have only a couple of suggested edits to phrasing that can be fixed in the final submission or even the proof stage.
1. Please use page numbers on manuscripts!
2. Section "The modified reference improves variant detection across ancestries". As a community we should move away from "European sample" etc towards "a sample with European ancestries". Any terminology is imperfect, but Graham Coop argues clearly that this might be the best we can get given the complexities of dealing with human genetic ancestry (arXiv:2207.11595).
3. Paragraph after Fgure 5 legend, second line. "play out" is a bit colloquial and unnecessarily confusing - should be "we saw this in our experiment".
4. In my review copy, Figure 6 was somewhat blurred and only just readable. Ensure that high resolution bitmap/vector graphic images are provided for publication.

**Reviewer 2**

The authors present FixItFelix, a tool designed to remove falsely aligned reads in existing read alignments generated on GRCh38, by extracting and re-aligning the reads in the known collapsed or falsely duplicated region. The authors created a modified GRCh38 reference using sequences from T2T-CHM13. The results compellingly show improvements in mapping, variant calling in genomic and/or expression data regardless of the sample's population, while reducing mapping artifacts that can result in artifactual cis-eQTLs.

However, the methods leads into a fundamental question of using T2T-CHM13 as the initial mapping target. I agree that re-doing the alignment could be expensive (not only compute time, also including manual labor for manipulating the alignment), however, given the additional burden one needs to spend for re-analyzing regions with mixed-results (i.e. DUSP22), or still has to ignore (i.e. mis-assemblies or gaps in GRCh38) it may be better to begin using T2T-CHM13 as the reference, then lifting the data over back to GRCh38 using tools such as levioSam2 (Chen et al, bioRxiv, 2022). This way, one can leverage both the rich annotation data bases available on GRCh38, and can go back to regions only available on T2T-CHM13 (e.g. corrected sequences with more complicated re-arrangements, such as the trans-location mis-assembly in LINC01150 ~ TNNT3 from Aganezov et al.). In fact, the F1 scores of the SNVs seems higher in results presented in levioSam2, although this is not an apple-to-apple comparison as the variant calling procedure may be slightly different. Therefore, I would argue that a proper comparison of using FixItFelix vs. levioSam2 needs to be made, and the advantage of using FixItFelix over levioSam2 needs to be discussed.

One additional minor comment: Fig. 2AB bar charts show "FixItFelix" result compared to the original mapping, while the main text is about comparing MQ in original vs. modified reference. I assume the MQ of using the modified reference and after applying FixItFelix are identical in the regions, if so please clarify in the legend and correct the color. However, if not, please add all 3 MQs to Fig. 2AB as in CD.

**Authors’ response**

We would like to thank the reviews for the suggestions and seeing the value in FixItFelix. Over the past weeks, we integrated their suggestions and have improved the manuscript accordingly. We now also added a section where we benchmark our approach to a recently available alignment lift over approach (Leviosam2) and we could show that our approach is overall improving the variant detection the most and in shorter runtime. To keep track of changes we highlighted our text in blue for the reviewer responses and changes in the manuscript itself. We have now also enclosed the Figures in high quality embedded in a PowerPoint slide deck.

**Reviewer #1**: Fixing errors in the reference assembly are an unfashionable yet very important part of genomics, as so much downstream analysis in medical genomics relies on, and will continue to rely on, mapping sequencing reads to a reference sequence and interpreting differences as genuine variation in the diploid genome of the individual sequenced. This paper is very welcome, as it addresses particular erroneous regions, provides an accessible software tool to fix existing sequence alignments, and demonstrates the importance of this by illustrating variant calling on medically-important genes in the fixed region.

Overall, this is an excellent paper. Clear to read, interesting, thorough and important. I have only a couple of suggested edits to phrasing that can be fixed in the final submission or even the proof stage.

1. Please use page numbers on manuscripts!

Thanks. We updated the page numbers in this version of the paper.

2. Section "The modified reference improves variant detection across ancestries". As a community we should move away from "European sample" etc towards "a sample with European ancestries". Any terminology is imperfect, but Graham Coop argues clearly that this might be the best we can get given the complexities of dealing with human genetic ancestry (arXiv:2207.11595).

Thanks for the important suggestion. We updated that in the revised manuscript.

3. Paragraph after Fgure 5 legend, second line. "play out" is a bit colloquial and unnecessarily confusing - should be "we saw this in our experiment".

Thanks. We updated that sentence.

4. In my review copy, Figure 6 was somewhat blurred and only just readable. Ensure that high resolution bitmap/vector graphic images are provided for publication.

We uploaded all images in high resolution.

**Reviewer #2:** The authors present FixItFelix, a tool designed to remove falsely aligned reads in existing read alignments generated on GRCh38, by extracting and re-aligning the reads in the known collapsed or falsely duplicated region. The authors created a modified GRCh38 reference using sequences from T2T-CHM13. The results compellingly show improvements in mapping, variant calling in genomic and/or expression data regardless of the sample's population, while reducing mapping artifacts that can result in artifactual cis-eQTLs.

However, the methods leads into a fundamental question of using T2T-CHM13 as the initial mapping target. I agree that re-doing the alignment could be expensive (not only compute time, also including manual labor for manipulating the alignment), however, given the additional burden one needs to spend for re-analyzing regions with mixed-results (i.e. DUSP22), or still has to ignore (i.e. mis-assemblies or gaps in GRCh38) it may be better to begin using T2T-CHM13 as the reference, then lifting the data over back to GRCh38 using tools such as levioSam2 (Chen et al, bioRxiv, 2022). This way, one can leverage both the rich annotation data bases available on GRCh38, and can go back to regions only available on T2T-CHM13 (e.g. corrected sequences with more complicated re-arrangements, such as the trans-location mis-assembly in LINC01150 ~ TNNT3 from Aganezov et al.). In fact, the F1 scores of the SNVs seems higher in results presented in levioSam2, although this is not an apple-to-apple comparison as the variant calling procedure may be slightly different. Therefore, I would argue that a proper comparison of using FixItFelix vs. levioSam2 needs to be made, and the advantage of using FixItFelix over levioSam2 needs to be discussed.

Thanks for the suggestion. This is really important to compare the performance of FixItFelix with levioSAM2.  Most of the analysis was done prior to the release of LevioSAM2 pre-print. Nevertheless, we have now included this benchmark in the main text.

Overall we observed that our method (FixItFelix) shows a better performance in GRCh38 falsely duplicated regions (F-scores: 0.980 for SNs, 1.0 for INDELs) compared to Leviosam (F-scores: 0.973 for SNVs, 0.583 for INDELs). For the collapsed regions we do see that Leviosam has a slight improvement (F-scores for FixItFelix: 0.177 for SNVs, 0.319 for INDELs , Leviosam: 0.180 for SNVs, 0.405 for INDELs).

As expected the runtime is longer for Leviosam. Here, to obtain the shortest runtime, we used FixItFelix to map the reads within the problematic regions to the T2T-CHM13 reference and then we used Leviousam2 to lift them back over. Overall this took two times longer resulting in a ~15 min runtime for Leviosam2 pipeline.

We give the detailed description and numbers in the supplementary for the readers and highlight these results in the main text.

One additional minor comment: Fig. 2AB bar charts show "FixItFelix" result compared to the original mapping, while the main text is about comparing MQ in original vs. modified reference. I assume the MQ of using the modified reference and after applying FixItFelix are identical in the regions, if so please clarify in the legend and correct the color. However, if not, please add all 3 MQs to Fig. 2AB as in CD.

Thanks for the comment. Yes, FixItFelix re-maps to the modified reference genome. We updated the text in the revised manuscript.

**2nd round**

**Reviewer 2**

The authors benchmarked FixItFelix results against LevioSAM2 as requested. I have a few additional questions and comments, all are minor.

The results were interesting, FixItFelix performed slightly better in false duplications while LevioSAM2 performed slightly better in collapsed regions. The authors indicate the increased false negatives with LevioSAM2 in false duplications resulted in the better performance of FixItFelix. What would cause the increased false negatives in using T2T-CHM13 as the reference, that are not observed in FixItFelix? A little more detailed explanation would be helpful to understand the major differences between the two approaches. The run time comparison method was fair, and results were as expected.

In addition, I was expecting some discussion about the limitations of FixItFelix. The authors generated a modified reference targeting false duplications and collapses errors in GRCh38 to “fix”, however there are many other errors not easily addressable in GRCh38 but could be addressed by using a more complete and accurate assembly (e.g. T2T-CHM13) as a reference. Calling variants accurately in the mis-assembled translocation example from my previous comments would be one such example, and of course, all variants in the newly added sequences that are impossible to map on the GRCh38 coordinates should be taken into consideration. I believe majority of the readers are already aware of this, however the overall message delivered from this manuscript could mislead some readers to conceive that this tool is an ultimate solution for all the shortcomings in GRCh38 and can completely replace the need of using the T2T-CHM13 references. Making a clear description of the limitations would be helpful for those readers to understand which errors are “not” expected to be fixed, and that the T2T-CHM13 should be considered as an alternate reference in such cases.

One thing I overlooked in my initial review was the results from the T2T Diversity Panel. The 8 individuals from the T2T Diversity Panel are part of the 1KGP samples, which is not widely known. The “draft benchmark set” was also confusing. Please clarify that variant calls generated with dipcall and hifiasm was “treated as a truth set” when performing the benchmark, and state that the variants called from (30X Illumina reads?) were benchmarked. Please provide the above information in the Results section. It took me some time to understand how the benchmark was performed.

My excel complained that “We found a problem with some content in 'GRCh38_manuscript_Supplementary_Tables.xlsx'.”. Please double check the supplementary tables file is properly containing all data.

**Authors’ response**

**Reviewer #2:** The authors benchmarked FixItFelix results against LevioSAM2 as requested. I have a few additional questions and comments, all are minor.

The results were interesting, FixItFelix performed slightly better in false duplications while LevioSAM2 performed slightly better in collapsed regions. The authors indicate the increased false negatives with LevioSAM2 in false duplications resulted in the better performance of FixItFelix. What would cause the increased false negatives in using T2T-CHM13 as the reference, that are not observed in FixItFelix? A little more detailed explanation would be helpful to understand the major differences between the two approaches. The run time comparison method was fair, and results were as expected.

Thanks for the suggestion. The false negatives of LevioSAM2 in falsely duplicated regions are indeed very interesting. As you know LevioSAM2 is doing a genome wide lift over of alignments and has thus a different objective compread to FixItFelix. We could speculate that this might be due to the chaining file or other things in LevioSAM2, but would point to the LevioSAM2 authors to address this more accurately.

In addition, I was expecting some discussion about the limitations of FixItFelix. The authors generated a modified reference targeting false duplications and collapses errors in GRCh38 to “fix”, however there are many other errors not easily addressable in GRCh38 but could be addressed by using a more complete and accurate assembly (e.g. T2T-CHM13) as a reference. Calling variants accurately in the mis-assembled translocation example from my previous comments would be one such example, and of course, all variants in the newly added sequences that are impossible to map on the GRCh38 coordinates should be taken into consideration. I believe majority of the readers are already aware of this, however the overall message delivered from this manuscript could mislead some readers to conceive that this tool is an ultimate solution for all the shortcomings in GRCh38 and can completely replace the need of using the T2T-CHM13 references. Making a clear description of the limitations

would be helpful for those readers to understand which errors are “not” expected to be fixed, and that the T2T-CHM13 should be considered as an alternate reference in such cases.

FixItFelix is clearly a targeted approach and requires prior identification of these regions. Thus applying it to other genome builds will be possible, but requires some steps before hand. Also as we mentioned some issues with genes can not be fully resolved with this approach. Nevertheless, we clearly provide evidence that FixItFelix is beneficial for sequencing analysis.

We now added in the discussion: “Other limitations of FixItFelix is that the program itself focuses on these regional approaches rather than a genome wide approach. This also includes that these regions need to be identified first based on genomic alignments and this might require some expertise. Thus applying it to other genome builds will be possible, but requires some steps before hand. “

One thing I overlooked in my initial review was the results from the T2T Diversity Panel. The 8 individuals from the T2T Diversity Panel are part of the 1KGP samples, which is not widely known. The “draft benchmark set” was also confusing. Please clarify that variant calls generated with dipcall and hifiasm was “treated as a truth set” when performing the benchmark, and state that the variants called from (30X Illumina reads?) were benchmarked. Please provide the above information in the Results section. It took me some time to understand how the benchmark was performed.

We added now a sentence. The result text reads as follows: “ We first generated the draft benchmark sets for these samples by calling variants with dipcall by using hifiasm assemblies of their paternal and maternal haplotypes. The Illumina data was then processed similarly than before and we compared the GATK variant performance before and after applying FixItFelix (see methods).”

My excel complained that “We found a problem with some content in 'GRCh38_manuscript_Supplementary_Tables.xlsx'.”. Please double check the supplementary tables file is properly containing all data.

This was downloaded from google drive. We checked the file and reuploaded it.
